# Development and validation of primary human myometrial cell culture models to study pregnancy and labour

**DOI:** 10.1186/1471-2393-13-S1-S7

**Published:** 2013-01-31

**Authors:** Andrea A Mosher, Kelly J Rainey, Seunghwa S Bolstad, Stephen J Lye, Bryan F Mitchell, David M Olson, Stephen L Wood, Donna M Slater

**Affiliations:** 1 Department of Physiology & Pharmacology, University of Calgary, Calgary, Alberta, Canada; 2 Samuel Lunenfeld Research Institute, Mount Sinai Hospital, University of Toronto, Toronto, Canada; 3 Departments of Physiology, Obstetrics & Gynecology, and Pediatrics, University of Alberta, Edmonton, Canada; 4 Department of Obstetrics & Gynaecology, University of Calgary, Alberta, Canada

## Abstract

**Background:**

The development of the *in vitro* cell culture model has greatly facilitated the ability to study gene expression and regulation within human tissues. Within the human uterus, the upper (fundal) segment and the lower segment may provide distinct functions throughout pregnancy and during labour. We have established primary cultured human myometrial cells, isolated from both upper and lower segment regions of the pregnant human uterus, and validated them for the purpose of studying human pregnancy and labour. The specific objectives of this study were to monitor the viability and characterize the expression profile using selected cellular, contractile and pregnancy associated markers in the primary cultured human myometrial cells. Labour has been described as an inflammatory process; therefore, the ability of these cells to respond to an inflammatory stimulus was also investigated.

**Methods:**

Myometrial cells isolated from paired upper segment (US) and lower segment (LS) biopsies, obtained from women undergoing Caesarean section deliveries at term prior to the onset of labour, were used to identify expression of; α smooth muscle actin, calponin, caldesmon, connexin 43, cyclo-oxygenase-2 (*COX-2*), oxytocin receptor, tropomyosin and vimentin, by RT-PCR and/or immunocytochemistry. Interleukin (IL)-1β was used to treat cells, subsequently expression of *COX-2* mRNA and release of interleukin-8 (CXCL8), were measured. ANOVA followed by Bonferroni’s multiple comparisons test was performed.

**Results:**

We demonstrate that US and LS human myometrial cells stably express all markers examined to at least passage ten (p10). Connexin 43, *COX-2* and vimentin mRNA expression were significantly higher in LS cells compared to US cells. Both cell populations respond to IL-1β, demonstrated by a robust release of CXCL8 and increased expression of *COX-2* mRNA from passage one (p1) through to p10.

**Conclusions:**

Isolated primary myometrial cells maintain expression of smooth muscle and pregnancy-associated markers and retain their ability to respond to an inflammatory stimulus. These distinct myometrial cell models will provide a useful tool to investigate mechanisms underlying the process of human labour and the concept of functional regionalization of the pregnant uterus.

## Background

Efforts to understand mechanisms regulating the balance between uterine quiescence and contractions are a main focus of research in obstetrics, especially preterm labour. Of particular interest are the myometrial smooth muscle cells as these constitute the contractile machinery of the uterus and may offer a therapeutic target for the prevention of premature myometrial contractions. Cell culture provides a valuable *in vitro* tool to study many human tissues and organs with the potential to gain insight into various physiological and pathological processes. However, there are concerns about the lifespan of cultured primary cells and their ability to continue to be representative of the tissue of origin. Therefore, we have established primary cultured human myometrial cells isolated from myometrial tissue biopsies obtained during elective Caesarean section deliveries at term, prior to the onset of labour, and validated their usefulness for studying pregnancy and labour.

It has been hypothesized that the upper and lower regions of the human uterus are functionally distinct [1]. During labour, the upper segment has a more contractile phenotype, contracting to push down and initiate delivery of the baby, whilst the lower segment, maintains a more relaxed phenotype, to facilitate delivery of the baby through the lower segment of the uterus and cervix. A number of genes, thought to be involved in the onset of labour, have been shown to be differentially expressed in upper and lower segment myometrial tissues [2-7]. These observations support the notion that upper and lower segments of the pregnant human uterus may be functionally distinct.

The objective of the current study was to establish primary cultures of human myometrial cells isolated from paired upper and lower segment myometrial biopsies for ten passages and evaluate and compare the expression of smooth muscle markers, fibroblast markers, contractile proteins or labour associated proteins over time. For the purpose of this study, smooth muscle markers were defined as; α smooth muscle actin, calponin, caldesmon, and tropomyosin, since α smooth muscle actin is part of the contractile machinery of smooth muscle cells and calponin, caldesmon, and tropomyosin are proteins that modulate muscle cell contractility through their ability to bind to α smooth muscle actin [8-10]. Fibroblast markers used were vimentin, a filament protein that is typically used to identify fibroblasts [11,12] and a fibroblast surface protein called IB10 (http://Abcam.com). The gap junction protein, connexin 43 and the oxytocin receptor are labour associated genes known to be up-regulated at the time of labour onset in human myometrium [3,5,13,14].

In addition, labour is commonly considered an inflammatory process and up-regulation of inflammatory genes including interleukin-1β (IL-1β), cyclo-oxygenase-2 (*COX-2*) and interleukin-8 (CXCL8) is observed [15-17,5]. We therefore tested the ability of the upper segment myometrial cells to respond to an inflammatory stimulus compared to lower segment cells. For this study, interleukin-1β (IL-1β) was used as the inflammatory stimulus, and cyclo-oxygenase-2 (*COX-2*) mRNA expression and interleukin-8 (CXCL8) release were the outcomes measured.

## Methods

### Sample collection

Institutional ethical committee approval was obtained (Conjoint Health Research Ethics Board, Office of Medical Bioethics at the University of Calgary) for the collection of myometrial biopsies to study the mechanisms of human labour. Women undergoing delivery by elective caesarean section at term were recruited, fully informed and consented to the study. In the current study, none of the women had any known underlying diseases, all were Caucasian with ages ranging from 25-41. Indications for caesarean delivery included breech presentation or previous caesarean delivery. Gestational age ranged from 38 weeks + 4 days to 39 weeks + 0 days and no clinical signs of labour were observed. Paired biopsies were collected from the lower and upper regions of the uterus following delivery of the infant. The lower segment biopsy was taken from the exposed myometrium on the upper aspect of the lower segment incision. The upper segment biopsies were all taken from the side opposite the placenta on the anterior or posterior aspect of the upper segment. Palpation and visualization of the uterus determined where the upper segment begins and samples were taken from approximately half way between the upper edge of the lower segment incision and the tip of the fundus. For the upper segment biopsy, the decidual layer was first dissected away and a small piece of myometrium grasped with fine forceps and dissected with Iris scissors. All samples were placed immediately into sterile cold Hank’s Balanced Salt Solution (HBSS) (Life Technologies, Burlington, ON, Canada), containing antibiotic-antimycotics (1x Penicillin-Streptomyocin-Amphotericin B) (Gibco, Life Tecnologies, Inc), and transported to the laboratory for myometrial cell isolation within 30 minutes of delivery.

### Isolation and culture of primary human myometrial cells

Myometrial cells were isolated, separately but in parallel, from the paired, upper segment (US) and lower segment (LS) myometrial biopsies. Myometrial cells were isolated from methods adapted from Phaneuf *et al*., [18] and Tribe *et al*., [19]. Each biopsy was dissected into 1 mm^3^ pieces, washed in HBSS to remove excess blood, placed in Smooth Muscle cell medium containing collagenase XI (1 mg/ml), collagenase IA (1 mg/ml) and fatty acid free bovine serum albumin (0.5%) (Sigma-Aldrich, Oakville, ON, Canada), and digested for 60 minutes at 37^o^C. Myometrial cells were then dispersed by passing the digestion solution repeatedly through a fine sterile pipette, further digestion was stopped by the addition of 5% fetal bovine serum (FBS) (Life Technologies) contained in Smooth Muscle Medium (PromoCell, Heidelberg, Germany). Cells were passed through a 70 μm sieve, centrifuged for 5 minutes at 400 g, and re-suspended in Smooth Muscle Medium containing 5% FBS and 1x antibiotic-antimycotic. Isolated cells were assessed for viability by the Trypan Blue exclusion method (Life Technologies Inc.) and counted using the Countess Automated Cell Counter (Life Technologies Inc.). Cells were plated into 25 cm^2^ culture flasks (Corning Inc., Lowell, MA, USA), incubated at 37^o^C in a humidified atmosphere of 95% air/5% CO_2_ and the culture medium replaced every 2 days. The initial stage of cell growth was termed passage 0 (p0). At approximately 10-14 days following initial isolation when myometrial cell growth reached 80-90% confluence (% coverage of cells in the culture flask) the cells were sub-cultured (passaged) and plated at a density of 4.0 x 10^4^ cells/ml into a larger 150cm^2^ culture flask and termed, passage 1 (p1).

For the current series of experiments LS and US myometrial cells were sub-cultured from passage 1 through to passage 10 at a density of 4.0 x 10^4^ cells/ml into a 150 cm^2^ culture flask and 12-well plates (Corning Inc.) to assess changes in; (i) cell viability and population doubling, (ii) expression of contractile markers (mRNA and protein) and, (iii) the response to treatment with the inflammatory mediator interleukin-1β (IL-1β). Prior to experiments, all cells were serum deprived overnight (Smooth Muscle Medium 0.5% FBS), then incubated for 6 hours with either 1 ng/ml IL-1β (Research & Development Systems, Minneapolis, MN, USA) or non-treated (NS). Culture supernatants were collected and stored at -20^o^C while cell culture plates were stored at -80^o^C prior to RNA isolation.

#### RNA isolation, conventional RT-PCR and real-time RT-PCR

Total RNA was isolated from human myometrial cells using the Qiagen RNeasy Mini Kit (Qiagen Inc., Toronto, ON, Canada) and 500 ng used for reverse transcription and synthesis of complementary DNA (cDNA) with qScript cDNA SuperMix (Quanta BioSciences Inc., Gaithersburg, MD, USA). Negative reverse transcription reactions were performed as a control, in which the reverse transcriptase enzyme was absent. Conventional RT-PCR was performed on an Eppendorf EPS Mastercycler (Eppendorf Canada, Mississauga, ON, Canada) with an initial denaturing step of 2 minutes at 94^o^C, followed by 32-36 cycles of denaturing at 94^o^C, annealing at 58^o^C -60^o^C, and elongation at 72^o^C, with a final elongation step at 72^o^C for 5 minutes. RT-PCR products were first analyzed by agarose gel electrophoresis to confirm the RT-PCR amplification product was the correct size. Subsequently, RT-PCR products were purified using a Qiagen MinElute PCR Purification Kit (Qiagen Inc.), and verified by sequencing (DNA Sequencing Services, University of Calgary). Real-time RT-PCR was performed on the Applied Biosystems 7900HT machine in 96-well Fast Optical MicroAmp Plates (Applied Biosystems, Life Technologies) with SYBR Green qPCR SuperMix (Quanta BioSciences Inc.). All protocols were performed according to manufacturer’s instructions. See Table 1 for PCR primer sequences. Melt curve analysis was performed to verify that non-specific amplification products were not present. Gene expression was normalized to the reference gene β2-microglobulin (B2M) and results calculated using the ΔΔC_T_ method [20].

**Table 1:** Primer sequences

Name	Primer Sequences (5’ – 3’)	Product Size Conventional Or real-time	***Target Gene*****(GenBank Accession)**
β2-microglobulin	F: CTTATGCACGCTTAACTATCTTAACAA R: TAGGAGGGCTGGCAACTTAG	127bp Real-time	*B2M* NM_004048

Calponin	F: GATGGCATCATTCTTTGCGA R: TTGTAGTAGTTGTGTGCGTG F: CTGAGAGAGTGGATCGAGGG R: TGATCTTCTTCACGGAGCCT	701bp Conventional 127bp Real-time	*CNN1* NM_001299

Connexin 43	F: GTGCCTGAACTTGCCTTTTC R: GATGATGTAGGTTCGCAGCA F: GGAGTTCAATCACTTGGCGT R: ACACCTTCCCTCCAGCAGTT	522bp Conventional 134bp Real-time	*GJA1* NM_000165

Cyclo-oxygenase-type 2 also known as Prostaglandin GH synthase-2	F: AGATCATCTCTGCCTGAGTATCTT R: TTCAAATGAGATTGTGGAAAAATTGCT F: GCTGGGCCATGGGGTGGACT R: CCTGCCCCACAGCAAACCGT	305bp Conventional 200bp Real-time	*COX-2 or PTGS-2* NM_000963

Glyceraldehyde-phosphatedehydroegnase	F: CCACCCATGGCAAATTCCATGGCA R: TCTAGACGGCAGGTCAGGTCCACC	598bp Conventional	*GAPDH* NM_002046

Vimentin	F: TCAGAGAGAGGAAGCCGAAA R: GTGAGGGACTGCACCTGTCT F: GGCTCAGATTCAGGAACAGCATG R: CCTGTCTCCGGTACTCAGTGGAC	380bp Conventional 230bp Real-time	*VIM* NM_003380

Sequence of primers used for conventional and real-time RT-PCR analysis.

### ELISA

The chemokine, CXCL8 was quantified by a sandwich enzyme-linked immunosorbent assay (ELISA) (Human DuoSet®, Research & Development Systems, Minneapolis, MN, USA) according to manufacturer’s instructions. Optical density was measured at 450 nm using a DTX880 Multimode Detector (Beckman Coulter Canada Inc., Mississauga, ON, Canada).

### Immunocytochemistry

Cells were also seeded onto 8-chamber glass culture slides (ThermoFisher Scientific, Ottawa, ON, Canada) at passage 1, passage 5 and passage 10 and grown to 80-90% confluence. Cells were rinsed in phosphate-buffered saline (PBS), fixed in 4% paraformaldehyde for 20 minutes at 37^o^C and permeabilized in 0.1% Triton-X in PBS for 15 minutes at room temperature. Myometrial cells were treated using a Dako kit (Dako Canada Inc., Burlington, ON, Canada) according to the manufacturer’s instructions. Briefly, cells were incubated for 10 minutes in 1.5% blocking serum in PBS at room temperature prior to incubation with primary antibody (Table 2). Cells were washed in PBS and incubated with biotinylated goat anti-rabbit and anti-mouse secondary antibody followed by peroxidase substrate. Finally, cells were counterstained in Harris’ haematoxylin and mounted with Dako Faramount Aqueous Mounting Medium. Images were obtained using a Zeiss Axio Scope connected to an Axiocam ICc 3 camera and AxioVision 4.8.2 software (Carl Zeiss Canada Ltd., BC, Canada).

**Table 2:** Antibody Information

Protein	Antibody information	Clonality	Dilution for ICC
α smooth muscle actin	sc-58669 (Santa Cruz)	Mouse monoclonal	1:1000

Caldesmon	ab32330 (Abcam)	Rabbit monoclonal	1:500

Calponin	ab46794 (Abcam)	Rabbit monoclonal	1:500

Oxytocin Receptor	sc-33209(Santa Cruz)	Rabbit polyclonal	1:200

Tropomyosin	sc-73225 (Santa Cruz)	Mouse monoclonal	1:50

Vimentin	sc-66001 (Santa Cruz)	Mouse monoclonal	1:1000

1B10	Ab11333 (Abcam)	Mouse monoclonal	1:500

#### Statistical analysis

All experiments were performed using paired upper and lower segment cells isolated from n=4 patients. All statistical analysis was performed with GraphPad PRISM Software (GraphPad Software Inc., San Diego, CA, USA). Graphical data are presented as the mean ± SEM. For comparison between two groups, Student's *t* test were used and for multiple comparisons a one-way analysis of variance (ANOVA) followed by post-hoc test of Bonferroni multiple comparisons. *P* < 0.05 was considered statistically significant.

## Results

**Figure 1:**
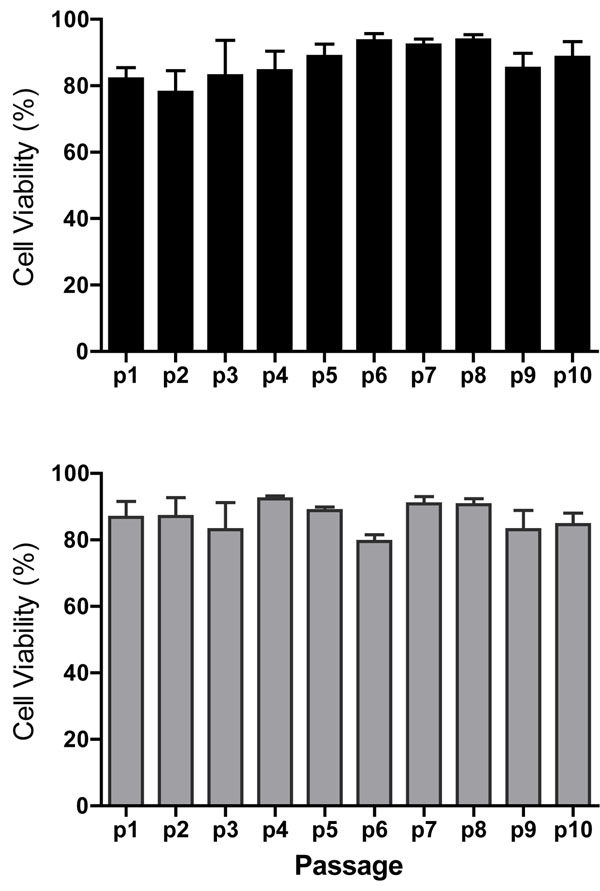
**Cell viability from p1 to p10** Viability (% live cells) of cultured upper segment (black bars) and lower segment (grey bars) myometrial cells at passage (p1 through p10). Values represent mean +/- SEM

Through the ten passages cell viability ranged from 78.0 - 93.75% and 79.5 - 92.5% in the US and LS cells respectively. There were no significant differences in cell viability through passage (p1 to p10) or when comparing cells isolated from the US versus the LS uterus as shown in Figure 1.

### Upper and lower segment human myometrial cells express smooth muscle and fibroblast markers

**Figure 2:**
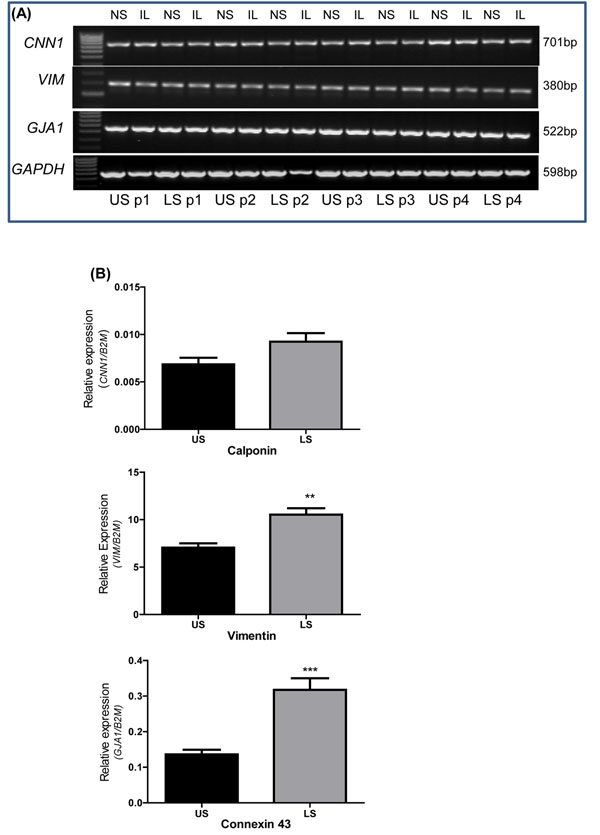
**Expression of calponin, vimentin and connexin-43 mRNA in upper and lower segment myometrial cells (A)** Representative agarose gel demonstrating expression of calponin (*CNN1*), vimentin (*VIM*), connexin 43 (*GJA1*) and *GAPDH* mRNA expression in upper and lower segment myometrial cells through passage 1 (p1) to passage 4 (p4) in non-treated (NS) and IL-1β (IL)-stimulated cells by conventional RT-PCR analysis. **(B)** Real-time RT-PCR analysis identified significantly higher expression of *VIM* and *GJA1* in LS myometrial cells (grey bars) compared to US cells (black bars). Data are presented as the mean ± SEM of all LS and US cells from p1 through p10 and analyzed by student *t* test. (** *P*=0.003, *** *P*<0.0001)

**Figure 3:**
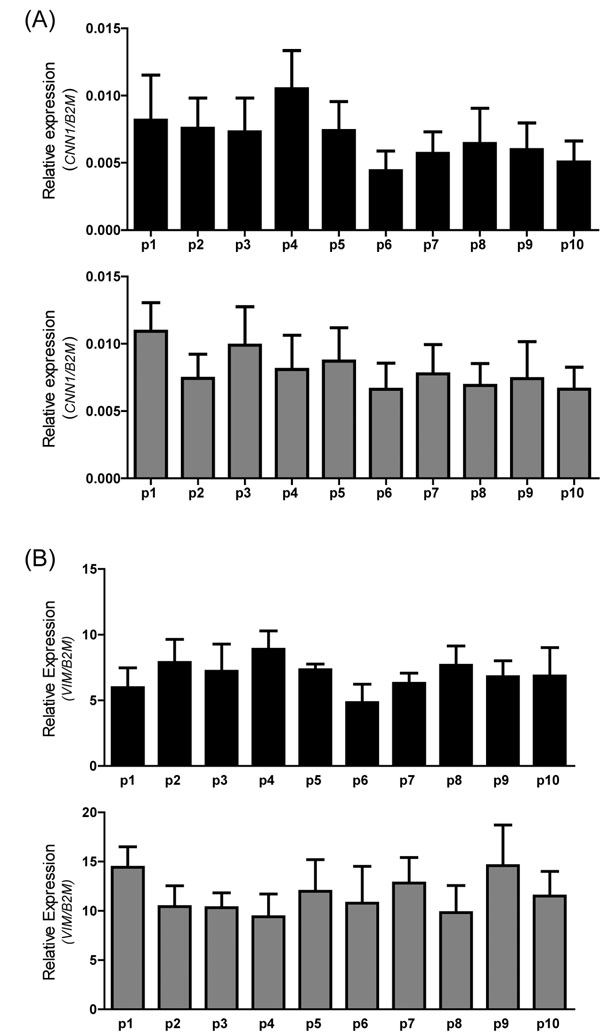
**Effect of passage (p1 through p10) on mRNA expression** Relative expression of (A) calponin (*CNN1*), and (B) vimentin (*VIM*) normalized to the reference gene β2-microglobulin (*B2M*). Data represent the mean ± SEM from US (black) and LS (grey) non-treated cells.

Total RNA was isolated from treated and non-treated upper and lower segment human myometrial cells (p1 to p10). Conventional RT-PCR was used to identify the presence of calponin (a smooth muscle marker; gene name *CNN1*), vimentin (a fibroblast marker; gene name *VIM*), and connexin-43 (a gap junction protein; gene name *GJA1*). *GAPDH* and *B2M* were used as reference housekeeping genes for conventional and real-time RT-PCR respectively. All four genes were positively identified in both upper and lower segment cells. *CNN1*, *VIM*, and *GJA1* mRNA expression were not affected by IL-1β treatment (Figure 2A). Qualitative data for p1 to p4 is shown in a representative agarose gel figure. The same trend was also observed for p5 to p10 (data not shown). Real-time RT-PCR was then used to quantify mRNA expression comparing upper and lower segment myometrial cells and to determine any changes through passage 1 to 10. Significantly higher expression of *VIM* (*P* = 0.032) and *GJA1* (*P* < 0.0001) but not *CNN1* (*P* = 0.06) was observed in lower segment cells (Figure 2B). Expression of *VIM*, *CNN1* (Figure 3A and B) or *GJA1* (data not shown) mRNA were not significantly different through p1 to p10.

### Upper and lower segment human myometrial cells differentially express COX-2 and respond to an inflammatory stimulus for at least ten passages

**Figure 4:**
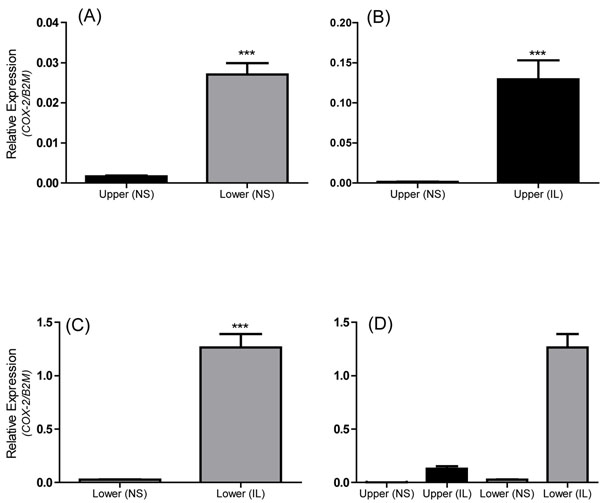
**Differential expression of COX-2 in LS versus US myometrial cells** Expression of COX-2 expression is significantly higher in the lower segment versus the upper segment in non-treated cells (A). Treatment with IL-1β significantly increases COX-2 expression in both upper segment (B) and lower segment (C) cells. Panel (D) shows for comparison the relative levels of COX-2 expression in all four groups. Relative expression of *COX-2* normalized to B2M. Data represent the mean ± SEM of US (black) and LS (grey) cells, analyzed by student *t* test through all passages (***P<0.0001)

To investigate the upper and lower segment human myometrial cells and the response of the cells to IL-1β, expression of cyclo-oxygenase (an inflammatory gene known to be increased during pregnancy and labour; gene name *COX-2*) mRNA was quantified by real-time RT-PCR and CXCL8 output was measured by ELISA. These outputs were chosen because both *COX-2* and CXCL8 are demonstrated to increase in human myometrium with labour [2,16]. Expression of COX-2 was first examined comparing upper segment versus lower segment cells, In non-treated cells expression of *COX-2* was significantly higher in lower segment cells compared to upper segment cells (*P* < 0.0001) (Figure 4A). Treatment with IL-1β significantly increased COX-2 expression in upper segment cells (Figure 4B) and lower segment cells (Figure 4C). Figure 4D demonstrates the relative expression of COX-2 as a direct comparison in upper, lower, non treated and IL-1β treated cells. IL-1β treatment results in increased expression of COX-2 in both upper and lower segment cells but the increase is much higher in the lower segment cells (Figure 4D).

**Figure 5:**
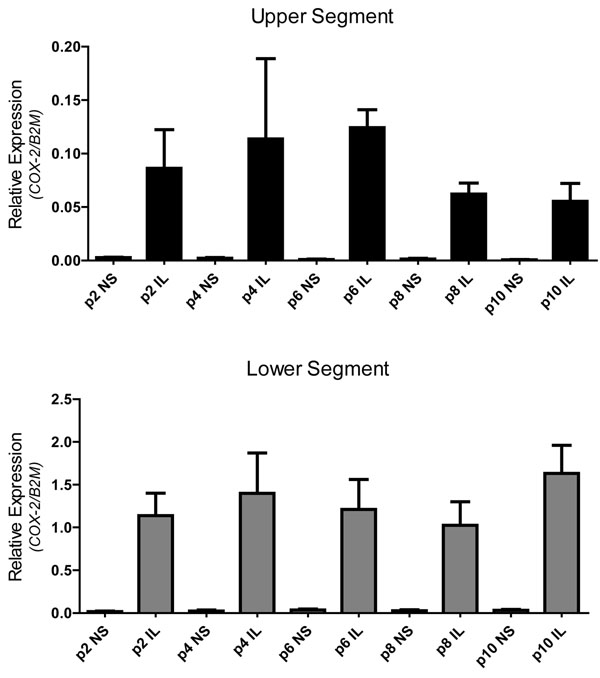
**IL-1β stimulates COX-2 mRNA expression in myometrial cells from p1 through p10** Relative expression of *COX-2* normalized to B2M. Data represent the mean ± SEM of US (black) and LS (grey) myometrial cells. For simplicity only data from p2, p4, p6, p8 and p10 are depicted in the graph. NS = non-treated cells; IL = IL-1β-stimulated cells. ANOVA (*P*=0.0007 in upper and *P*<0.0001 in lower segment).

**Figure 6:**
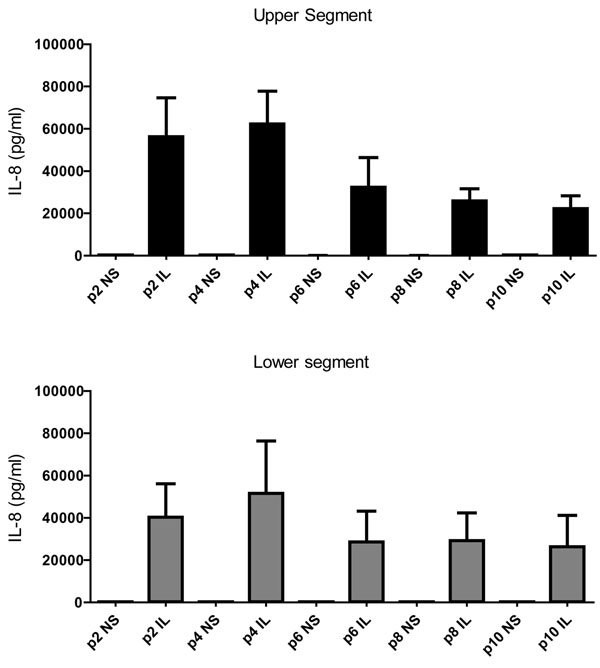
**IL-1β-induced CXCL8 output from p1 through p10** IL-1β stimulates release of CXCL8 from both upper (black) and lower (grey) segment myometrial cells Paired upper and lower segment human myometrial cells were non-treated (NS) or treated with IL-1β (IL). The culture supernatant was collected and CXCL8 output was quantified by ELISA. Data represent the mean ± SEM. Only data from p2, p4, p6, p8 and p10 are depicted in the graph. ANOVA (*P*<0.0001 in upper and *P*=0.0013 in lower segment).

Comparing passage number, treatment with IL-1β resulted in a robust increase in *COX-2* mRNA expression compared to the non-treated in both upper (ANOVA *P*=0.0007) and lower (ANOVA *P*<0.0001) segment cells (Figure 5). Treatment with IL-1β also resulted in a robust release CXCL8 (20 000-50 000 pg/ml) in upper (ANOVA *P*<0.0001) and lower (ANOVA *P*=0.0013) segment cells from p1 through p10 (Figure 6). There were no changes in the response to Il-1β from p1 through p10. Therefore, both upper and lower segment human myometrial cells retain their ability to respond to an inflammatory stimulus as demonstrated by increased expression of the inducible *COX-2* gene and increased release of the pro-inflammatory chemokine CXCL8.

### Upper and lower segment human myometrial cells stain positively for smooth muscle markers, fibroblast protein markers and contractile proteins

**Figure 7:**
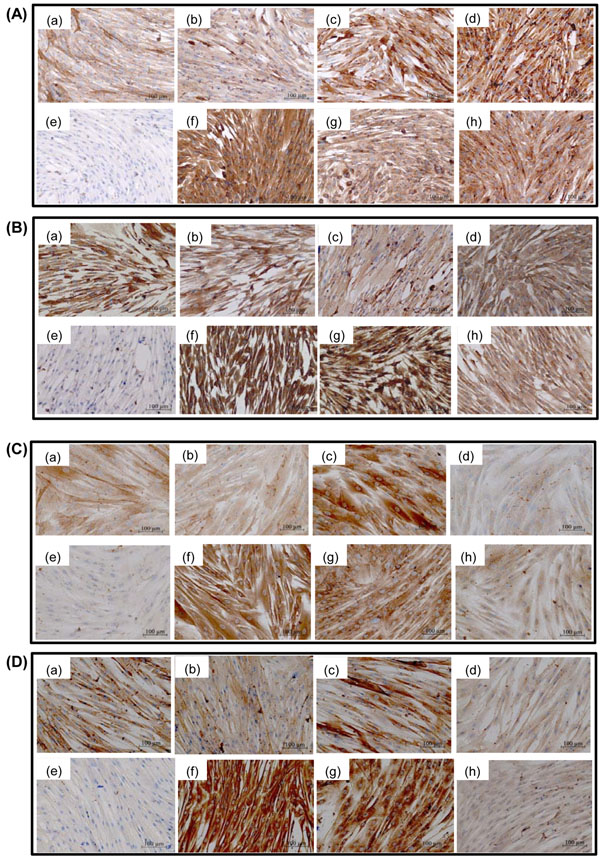
**Expression of smooth muscle markers, fibroblast markers and contractile proteins in upper and lower segment myometrial cells** Immunocytochemistry of (a) α smooth muscle actin, (b) caldesmon, (c) calponin, (d) tropomyosin, (f) Vimentin, (g) IB10, and the (h) oxytocin receptor in; (A) upper segment myometrial cells at p2, (B) lower segment myometrial cells at p2, (C) upper segment myometrial cells at p10 and (D) lower segment myometrial cells at p10. Negative controls were incubated without primary antibody (e).

Human myometrial cells were seeded on 8-chamber culture slides at passage 2, passage 5 and passage 10 and treated with antibodies to identify protein expression of smooth muscle markers, fibroblasts markers, and the oxytocin receptor (a contractile protein expressed in the pregnant uterus). Upper and lower segment human myometrial cells stained positively for α smooth muscle actin, caldesmon, calponin, tropomyosin, the oxytocin receptor, vimentin and 1B10 at p2, p5 and p10. On the whole similar staining patterns were observed at p2, p5 and p10, it was noted that staining for both tropomyosin and the oxytocin receptor appeared to be lighter at p10 compared to p2 however quantitative and statistical analysis was not performed due to the qualitative nature of the technique. (Figure 7) shows representative slides from (A) p2 and (B) p10.

## Discussion

In this study, we have shown that primary cells isolated and cultured from paired myometrial biopsies are viable and responsive for at least ten passages. Upper and lower segment human myometrial cells continue to grow, divide and respond to an external stimulus. From p1 through to p10, upper and lower segment cells respond to IL-1β treatment measured by increased gene expression (*COX-2*) and pro-inflammatory chemokine release (CXCL8).

Human myometrial cells are elongated, spindle-shaped cells and this morphology is maintained up to p10 with no discernible alterations in cell shape, size, or structure, as assessed by light microscopy. Cultured human myometrial cells expressed both smooth muscle markers (α smooth muscle actin, calponin, caldesmon, tropomyosin) and fibroblast markers (vimentin, 1B10), suggesting that they may represent an intermediate myofibroblast phenotype or possibly a mixed population of myometrial smooth muscle cells and myofibroblasts. However, another possibility is that the presence of myofibroblast type cells is due to the nature of the cell culture technique. Future work will seek to validate this and compare expression of myofibroblast markers within myometrial tissues. Cardiac myofibroblasts stain positively for both α smooth muscle actin and vimentin [21] and since the cultured human myometrial cells express both proteins, this evidence could suggest that they may be myometrial myofibroblasts. Myofibroblasts are involved in wound healing, remodeling of the extracellular matrix, as well as the development of fibrosis in pathological states [22,23]. For example, exposure to allergens can increase the number of myofibroblasts in the lung [24]. Myofibroblasts may originate by differentiation of fibroblast cells or de-differentiation of smooth muscle cells [21-23]. Thus, with the extensive growth and remodeling that occurs in the uterus during pregnancy, along with the restoration to the non-pregnant size and function following delivery, it may not be surprising, that the pregnant uterus could contain myofibroblasts.

An early report establishing human myometrial cells in culture found that the cells were stable for up to one year with no morphological changes [25]. However, there is concern that the lifespan of primary cultured cells is finite and that they eventually become senescent and unresponsive. A phenomenon known as the Hayflick Limit proposes that cell populations are capable of dividing for a fixed number of times before reaching senescence [26]. To address the limited lifespan of primary cells in culture immortalized cells lines have been produced either by transfecting telomerase expression vectors into primary cells [27] or transforming cells with the human papillomavirus or the SV-40 large T antigen [28-30]. It has been suggested that cell manipulation to achieve immortalization may cause a fundamental change in the growth of the cells that is no longer truly representative of the tissue of origin [31], calling into question whether such immortalized cell lines are truly representative of the tissue of origin, while other studies have reported that introduction of telomerase does not necessarily produce ‘cancer-like’ behaviour or ‘malignant’ properties in immortalized cells [32,33].

Ten years ago, non-pregnant human myometrial cells were immortalized using telomerase and investigated for their ability to retain characteristics of the myometrium. The telomerase-immortalized cells did express smooth muscle markers (α smooth muscle actin, calponin, caldesmon) but the study did not investigate the presence of fibroblast markers within the cells. The authors report that primary non-pregnant myometrial cells senesced after about 8 weeks in culture whereas the immortalized cells were still actively dividing after 10 months [9]. The concern about immortalized cells versus primary cells still persists, and many previous studies, including our own, have used primary cells only at low passages, typically p5 and lower [34], thus obtaining a continued supply of myometrial biopsies can be rate-limiting for the research. However, our data demonstrate that these cells can be maintained in culture for longer periods (to at least p10 as demonstrated) without loss of viability or response to IL-1β, for example. Condon *et al*., [9] also demonstrated that the immortalized cells maintained their ability to respond to oxytocin, which we have also shown in primary myometrial cells as oxytocin continued to stimulate calcium release by primary cells [35].

An additional area to explore would be to compare passaged cells with freshly isolated cells (p0) and/or tissue -explants which is a limitation of the current study. One of the difficulties is the ability to obtain sufficient fresh human myometrial tissue to perform the studies. However, based on the current data work is ongoing within our laboratory to develop additional models of myometrial tissue explants.

Comparison of our paired upper and lower segment myometrial cells (each pair being isolated from biopsies of the same uterus but distinct regions) revealed a number of clear differences. For example, *COX-2* mRNA expression was significantly greater in lower segment myometrial cells compared to upper segment cells (Figure 4), which is consistent with previously reported data from myometrial tissues [5-7]. Regional differences in gene expression have also been reported for the oxytocin receptor [3,7], connexin 43 [5,7], and the prostaglandin E_2_ receptors [4,2]. In addition, we show connexin 43 and vimentin mRNA expression is significantly higher in lower segment cells compared to the upper segment myometrial cells (Figure 2B). In contrast, calponin mRNA expression was not significantly different between upper or lower cells. Taken together, these observations support the concept of a functional regionalization of the upper and lower segment of the human uterus. Of importance to note is that the significant difference in COX-2 expression between upper and lower segment cells (Figure 4A) that is also maintained from p1 through p10 (Figure 5). Both the upper and the lower segment cells maintain the ability to respond to IL-1β treatment in terms of increased COX-2 expression. However, expression of COX-2 is already significantly higher in lower segment cells compared to the upper segment cells, this is in keeping with the expression pattern observed in myometrial tissues [5]. The ability to specifically isolate and culture both upper and lower segment human myometrial cells will aid investigation of the hypothesis of functional regionalization of the pregnant uterus and provide valuable knowledge about the mechanism underlying the process of human labour.

In summary, the results of this study advocate the use of primary cultured human myometrial cells to study the expression and regulation of pregnancy- and labour-associated genes within the human uterus. Human myometrial cells appear to retain molecular memory of their origin and maintain their ability to respond to the inflammatory stimulus IL-1β for at least ten passages. Moreover, these cells are not subject to additional manipulations in order to immortalize a cell phenotype. Future work will include further molecular and functional characterization of the myometrial cell subtypes within the human uterus.

## Conclusions

Primary human myometrial cells isolated from two distinct regions of the pregnant uterus offer a useful model for the study of regional functional differences and mechanistic pathways involved in the onset of human labour.

## List of abbreviations

B2M: beta 2 microglobulin; CNN1: calponin; COX-2: cyclo-oxygenase-2; CXCL8: interleukin 8; GAPDH: glyceraldehyde 3-phosphate dehydrogenase; GJA1: connexin 43; IL-1β: interleukin 1β; LS: lower segment; US: upper segment; VIM: vimentin.

## Competing interests

The authors declare that they have no competing interests.

## Authors' contributions

AAM, KJR, SJL, BFM, DMO, SLW and DMS conceived the study. SLW and DMS obtained ethical approval of the study. SLW obtained written and informed consent for all patients and performed all biopsy collections. DMS, AAM, and KJR isolated, cultured and treated cells. AAM, SSB and KJR performed the molecular, biochemical and immunocytochemical assays. AAM, KJR and DMS performed the data analysis. AAM and DMS drafted the manuscript. All authors read and approved the final manuscript.

## Declarations

This article has been published as part of *BMC Pregnancy and Childbirth* Volume 13 Supplement 1, 2013: Preterm Birth: Interdisciplinary Research from the Preterm Birth and Healthy Outcomes Team (PreHOT). The full contents of the supplement are available online at http://www.biomedcentral.com/bmcpregnancychildbirth/supplements/13/S1.

All of the publication fees will be funded by the Preterm Birth and Healthy Outcomes Team Interdisciplinary Team Grant (#200700595) from Alberta Innovates - Health Solutions, formerly the Alberta Heritage Foundation for Medical Research.
